# Third-line treatment and ^177^Lu-PSMA radioligand therapy of metastatic castration-resistant prostate cancer: a systematic review

**DOI:** 10.1007/s00259-017-3895-x

**Published:** 2017-12-16

**Authors:** Finn Edler von Eyben, Giandomenico Roviello, Timo Kiljunen, Christian Uprimny, Irene Virgolini, Kalevi Kairemo, Timo Joensuu

**Affiliations:** 1 Center of Tobacco Control Research, Birkevej 17, DK 5230 Odense M, Denmark; 2 Department of Oncology, Medical Oncology Unit, San Donato Hospital, Arezzo, Italy; 3 Department Medical, Surgery, and Health Sciences, University of Trieste, Trieste, Italy; 4 Docrates Cancer Center, Helsinki, Finland; 5 Department of Nuclear Medicine, University Hospital Innsbruck, Innsbruck, Austria

**Keywords:** Prostate cancer, ^177^Lu-PSMA radioligand therapy, Abiraterone, Enzalutamide, Docetaxel, Cabazitaxel, Systematic review

## Abstract

**Aims:**

There is a controversy as to the relative efficacy of ^177^Lu prostate specific membrane antigen (PSMA) radioligand therapy (RLT) and third-line treatment for patients with metastatic castration-resistant prostate cancer (mCRPC). The aim of our systematic review was to elucidate whether ^177^Lu-PSMA RLT and third-line treatment have similar effects and adverse effects (PROSPERO ID CRD42017067743).

**Methods:**

The review followed Preferred Reporting Items for Systematic Reviews and Meta-Analysis (PRISMA) guidelines. Searches in Pubmed and Embase selected articles up to September 2017. A search in ClinicalTrials.gov indicated ongoing studies. The meta-analysis used the random-effects model.

**Results:**

Twelve studies including 669 patients reported ^177^Lu-PSMA RLT. Overall, 43% of the patients had a maximum decline of PSA of ≥50% following treatment with ^177^Lu-PSMA RLT. The treatment with ^177^Lu-PSMA-617 and ^177^Lu-PSMA for imaging and therapy (I&T) had mainly transient adverse effects. Sixteen studies including 1338 patients reported third-line treatment. Overall, 21% of the patients had a best decline of PSA of ≥50% following third-line treatment. After third-line treatment with enzalutamide and cabazitaxel, adverse effects caused discontinuation of treatment for 10% to 23% of the patients. ^177^Lu-PSMA RLT gave a best PSA decline ≥50% more often than third-line treatment (mean 44% versus 22%, *p* = 0.0002, *t* test). ^177^Lu-PSMA RLT gave objective remission more often than third-line treatment (overall 31 of 109 patients versus 43 of 275 patients, *p* = 0.004, χ^2^ test). Median survival was longer after ^177^Lu-PSMA RLT than after third-line treatment, but the difference was not statistically significant (mean 14 months versus 12 months, *p* = 0.32, *t* test). Adverse effects caused discontinuation of treatment more often for third-line treatment than for ^177^Lu-PSMA RLT (22 of 66 patients versus 0 of 469 patients, *p* < 0.001, χ^2^ test).

**Conclusions:**

As for patients with mCRPC, treatment with ^177^Lu-PSMA-617 RTL and ^177^Lu-PSMA I&T gave better effects and caused fewer adverse effects than third-line treatment.

## Introduction

Prostate cancer (PC) is the most frequent non-cutaneous cancer and the second most frequent cause of cancer deaths for adult men. A worldwide estimate of PC in 2008 implied 899,000 new cases and 258,000 PC deaths [1]. Most patients with PC who die, die of metastatic PC (mPC) [2]. Six drugs increase overall survival for patients with metastatic castration-resistant prostate cancer (mCRPC) [3–8]. Patients with symptomatic mCRPC have initially been treated with docetaxel [3, 9]. Abiraterone, enzalutamide, cabazitaxel, sipoleucel, and ^223^radium increase overall survival for patients who had failed treatment with docetaxel [4–8, 10]. However, randomized trials have not evaluated the drugs for patients with failure in response to second-line treatment following recurrence after docetaxel. Therefore, European Association of Urology (EAU)/European Society of Radiotherapy and Oncology (ESTRO) guidelines do not recommend third-line treatment of mCRPC [11]. Due to unmet needs, the St. Gallen Advanced Prostate Cancer Consensus Conference (APCCC) 2017 gathered a representative group of experts for summarizing their opinions about treatment of advanced PC [12]. APCCC 2017 favored third-line treatment with cabazitaxel and with androgen receptor (AR) and AR signaling inhibitors.

Of PC, poorly differentiated, metastatic, and hormone-refractory adenocarcinomas of the prostate express prostate-specific membrane antigen (PSMA) [13]. ^68^Ga-PSMA HBED-CC PET/CT detects sites of cancer lesions for most patients with mCRPC [14, 15]. Patients with a positive ^68^Ga-PSMA HBED-CC PET/CT might be treated with ^177^Lu-PSMA radioligand therapy (RLT) [16]. ^177^Lu-J591 is a macromolecular radiolabeled humanized monoclonal antibody that targets the extracellular part of PSMA. ^177^Lu-J591 has a modest effect and causes frequent serious myelosuppression. ^177^Lu-PSMA-617 and ^177^Lu-PSMA I&T are small-molecule inhibitors of PSMA that give better effects and cause less adverse effects than ^177^Lu-J591.^117^Lu-PSMA RLT is mainly used as a compassionate treatment of patients with end-stage mCRPC [17]. For a patient with only lymph node metastatic CRPC, ^177^Lu-PSMA-617 RLT reduced PSA more than salvage radiotherapy and abiraterone [18]. In contrast, APCCC 2017 did not refer to ^177^Lu-PSMA RLT [19].

The discrepancy motivated us to carry out a systematic review comparing the two types of treatment [20]. The null hypothesis for our analyses was that ^177^Lu-PSMA RLT and third-line treatment of mCRPC have similar effects. The PROSPERO database registered our systematic review as CRD42017067743.

## Material and methods

Our systematic review evaluated the null hypothesis by comparing outcome following the two types of treatment.

## Search strategy

The systematic review followed guidelines of the Preferred Reporting Items for Systematic Reviews and Meta-Analysis (PRISMA) [21]. We selected articles that reported patients with mCRPC given ^177^Lu-PSMA RLT or third-line treatment and evaluated at least one effect measure. Reviewers undertook searches in Pubmed and Embase for articles published until September 2017. Two reviewers (FEvE and IV) searched independently for articles that reported ^177^Lu-PSMA RLT. A Pubmed search combined MESH terms and free text words: {(“prostat* neoplasm* [Mesh] OR prostate cancer) AND (prostate specific membrane antigen [Mesh] OR PSMA) AND (*lutetium [Mesh] OR *lu)}. The reviewers undertook a similar search in Embase. Two reviewers (FEvE and GR) searched independently for articles that reported third-line treatment. A Pubmed search combined MESH terms and free text words: {(“prostat* neoplasm* [Mesh] OR prostate cancer) AND (abiraterone [Mesh] OR enzalutamide [Mesh] OR cabazitaxel [Mesh]) AND (third line treatment OR third line therapy)}. The reviewers undertook a similar search in Embase. We used previous systematic reviews as external validation of our literature searches [16, 22, 23]. A reviewer (FEvE) also undertook a manual search and also a search for ongoing studies in ClinicalTrials.gov.

### Study selection

As regards ^177^Lu-PSMA RLT, we selected original research articles that reported ≥10 patients treated for mCRPC. Of several articles from a single center or a group of centers, we included the articles that reported the most patients. However, if the second of two articles from a center evaluated >50% of the patients who were not reported in the first article, we included both articles. We excluded articles that reported only biodistribution or dosimetry of ^177^Lu-PSMA RLT, and articles that used therapy with radioligands other than ^177^Lu-PSMA.

As regards third-line treatment, we selected original research articles that reported ≥10 patients treated for mCRPC. We selected articles of first- to third-line treatments that used only life-prolonging drugs. We included an article by Caffo et al. [24] that reported different sequences of drugs for the second- and third-line treatment. We also included an article by Brasso et al. [25] that summarized four previous articles of enzalutamide. Further, we included an article that reported cabazitaxel for patients who previously had failed with an AR inhibitor or an AR signaling inhibitor. Of articles that combined second- and third-line treatments or third- and fourth-line treatments, we included the articles that reported the third-line treatment separately. We excluded articles that reported only adverse effects.

## Data extraction

Of data from the selected articles, we extracted baseline characteristics such as year of publication, name of the first author, number of patients, and numbers of patients with metastases in lymph nodes, bones, and visceral organs. In the articles, surgical or medical castration implied serum testosterone was reduced to levels <50 ng/dL or <1.7 nmol/L. Hence, patients had CRPC if they had progression of PC despite castration levels of testosterone. We extracted treatment characteristics from articles of ^177^Lu-PSMA RLT such as number of previous treatments of mCRPC, median/mean PSA at start of ^177^Lu-PSMA RLT, type of ^177^Lu-PSMA RLT, median/mean number of cycles of treatment, median/mean interval between cycles, and median/mean administered activity of ^177^Lu for each cycle. We extracted treatment characteristics in articles of third-line treatment such as the drugs used as first-, second-, and third-line treatment, median/mean PSA at start of the third-line treatment, and dosage of the third-line drug. We extracted data on the frequency of severe adverse effects as graded by the National Cancer Institute Common Terminology Criteria for Adverse Events (CTCAE) version 4 for grade 3 and 4 hematologic and non-hematologic adverse effects.

The articles followed guidelines 2 by the Prostate Cancer Trials Working Group (PCWG2) [26]. As treatment endpoints in the articles, we extracted the frequency of best PSA decline of ≥50%, the frequency of objective response, and overall survival. The articles classified objective response by Response Evaluation Criteria in Solid Tumor (RECIST) 1.1 [27]. We combined complete remission (CR) and partial remission (PR) as objective remission. The articles defined overall survival as survival from start of treatment to death of any cause or to end of follow-up.

The selection of articles served as quality control.

A reviewer (FEvE) contacted principal authors for complementary information of selected articles.

## Statistical analysis

We undertook patient-based evaluations for each study and used parametric and non-parametric statistics in our evaluations. The articles calculated the frequency of treatment response as the proportion of responders of all patients. For articles with more than one response evaluation, we selected the highest frequency of response. The articles calculated the frequency of serious adverse effects as the proportion of patients with grade 3 to 4 adverse effect of all patients. We used the random-effects model in our meta-analysis because we assumed patients and treatments had hidden heterogeneity. We undertook funnel plots of the articles with the two types of treatment to evaluate articles for publication bias [28]. The meta-analysis generated forest plots of the articles to summarize the frequency of a best PSA decline of ≥50%. Forest plots were based on the software program metaprop for STATA, as described previously [29]. The metaprop analyses were based on the random-effects model. As for overall survival, we calculated the median and the interquartile range for the median overall survival reported in the articles. We used χ^2^ tests as we compared proportions of frequencies in the two groups of treatments and *t* tests as we compared distributions of frequencies. We considered a *p* value <0.05 as statistically significant.

One author (FEvE) performed all statistical analyses using the software STATA 14.2 (StataCorp., College Station, TX, USA).

## Results

### Studies of ^177^Lu-PSMA RLT

**Fig. 1:**
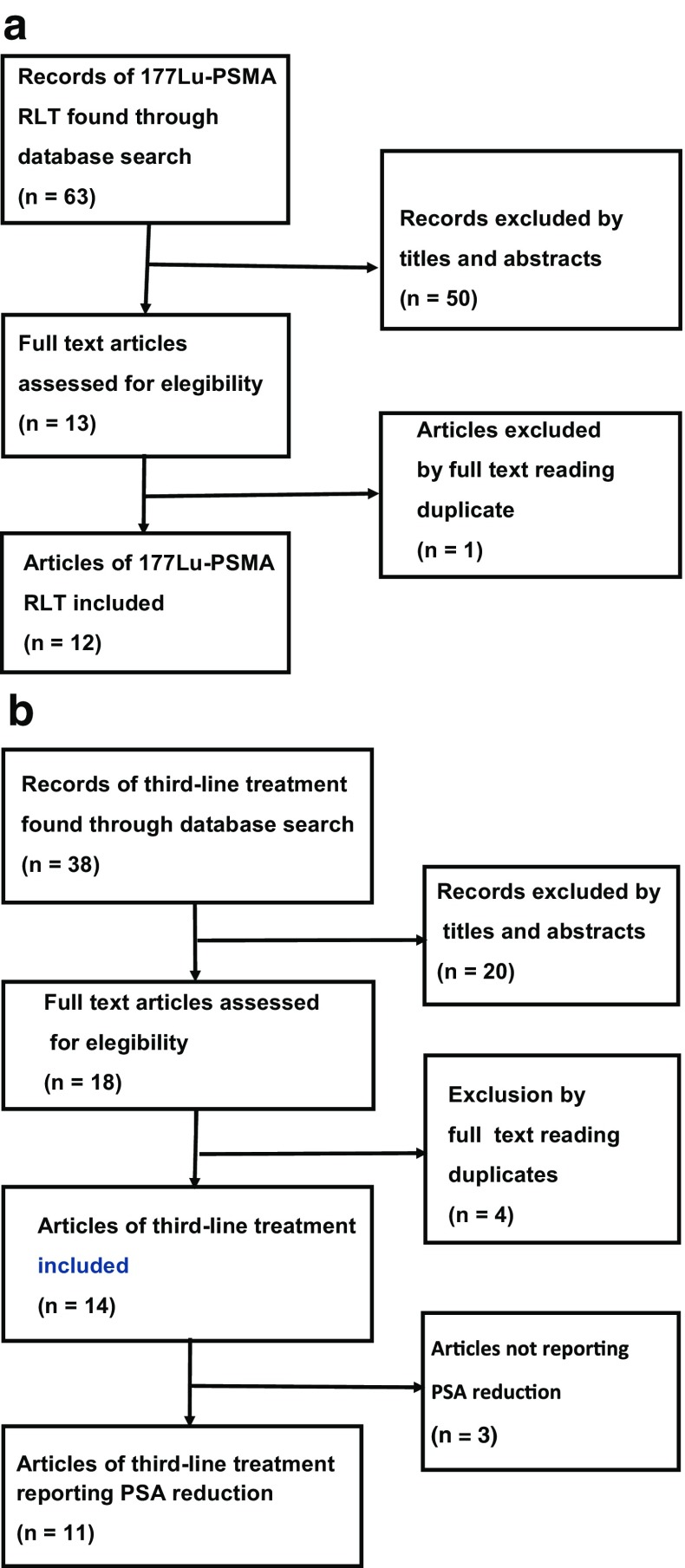
**a** Flow chart of the selection process for articles with ^177^Lu-PSMA RLT. **b** Flow chart of the selection process for selection of articles with third-line treatment

A search for articles of ^177^Lu-PSMA RLT gave 63 hits. The selected 12 articles consisted of 669 patients (Fig. 1a and Table 1) [30–41]. The median of the median/mean age in the articles was 70 years [25% and 75% interquartile range (IQR) 69–71 years]. The median of the median/mean pretreatment PSA was 130 ng/ml (IQR 77–306 ng/ml). Ten articles reported patients with end-stage mCRPC and two articles reported a heterogeneous group of patients [34, 40]. Nine articles used ^177^Lu-PSMA-617 RLT, two articles used ^177^Lu-J591 [30, 31], and one article used ^177^Lu-PSMA I&T [32]. A third of the patients underwent one cycle, the second third underwent two cycles, and the last third underwent three or more cycles. Nine articles administered ^177^Lu activity of approximately 6 G becquerel (GBq) for each cycle. During the study period, one article increased the administered ^177^Lu activity from 1.1 GBq to 6.0 GBq [37], one article from 4 to 6 GBq [33], and one article increased the administered activity from 3.7 to 7.4 GBq [32].

**Table 1:** Findings in articles of ^177^Lu-PSMA RLT (*n* = 12)

Author	No. of pts	Characteristics		Treatment				Outcome		
		Median age (yr)	Median pretreatment PSA (ng/ml)	Radioligand	No of cycles			Frequency of best PSA decline of ≥50% (%)	Frequency of objective remission (%)	Median overall survival (mos)
					*1*	*2*	*3*			
Bander [30]	35	68	30	J519	19	16	0	11 (4/35)	NR	NR
Tagawa [31]	47	74	74	J519	47	0	0	11 (5/47)	8 (1/12)	17
Heck [32]	22	71	349	I&T	9	5	8	33 (6/18)	5 (1/19)	NR
Kratochwil [33]	30	73	NR	617	1	18	11	43 (13/30)	NR	NR
Kulkarini [34]	119	71	NR	617	25	25	69	58 (46/80)	29 (17/58)	70% at 15 months
Ahmadzade far [35]	52	71	194	617	0	0	52	60 (31/52)	NR	14
Brauer [36]	59	72	346	617	13	0	46	53 (24/41)	NR	8
Fendler [37]	15	73	388	617	0	15	0	47 (7/12)	27 (4/15)	NR
Rahbar [38]	145	73	214	617	71	8	26	49 (49/99)	NR	NR
Rahbar [39]	104	70	361	617	NR	NR	NR	33 (34/104)	NR	14
Scarpa [40]	10	64	NR	617	0	1	9	50 (5/10)	30 (3/10)	NR
Yadav [41]	31	65	275	617	1	26	4	71 (22/31)	82 (5/6)	16
Total/median	669									

Abbreviations:

*NR* not reported, J519: ^177^Lu-J519, 617: ^177^Lu-PSMA-617, I&T: ^177^Lu-PSMA I&T, Objective remission: complete remission and partial remission

Overall, the articles reported the frequency decline of best PSA ≥50% for 1687 of 2007 (84%) of the patients.

### Efficacy

**Fig. 2:**
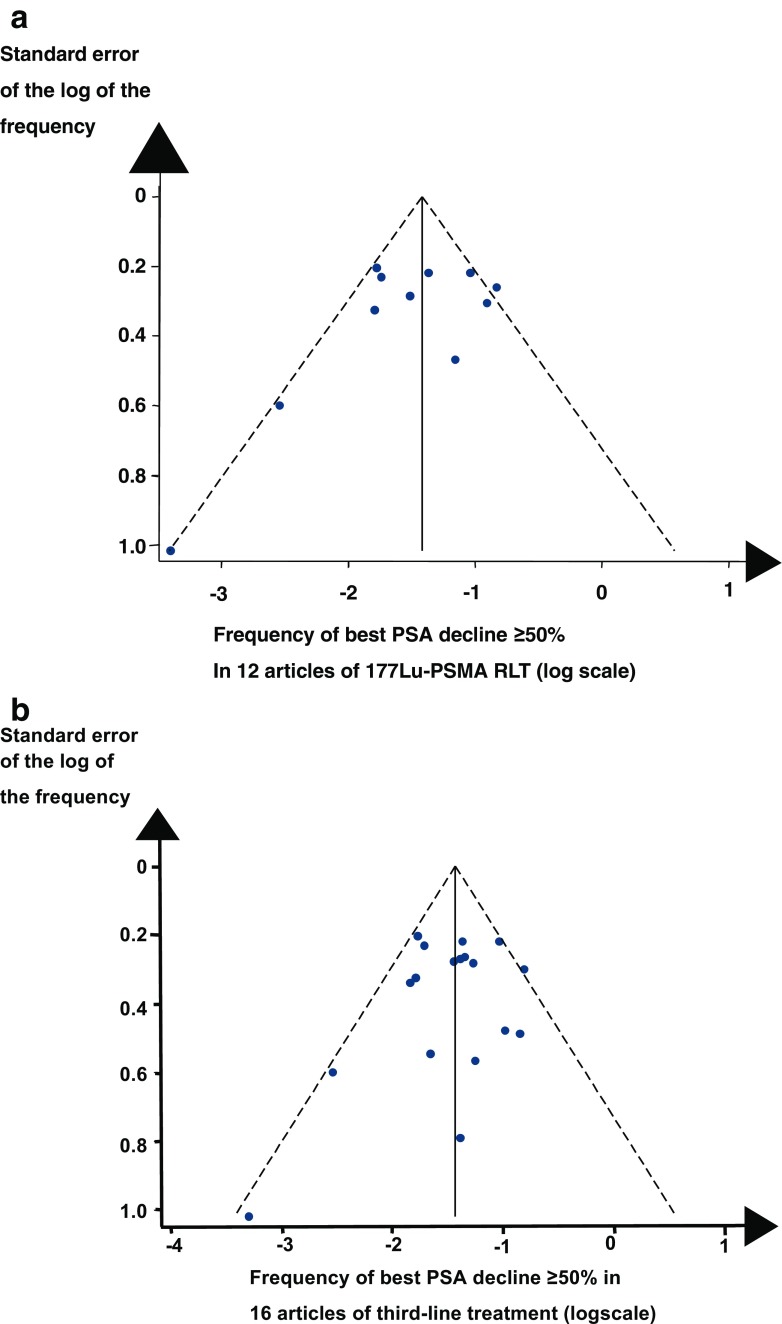
**a** Funnel plot of a best PSA decline of ≥50% in articles of ^177^Lu-PSMA-617 and ^177^Lu-PSMA I&T. **b** Funnel plot of a best PSA decline ≥50% in evaluable articles of third-line treatment

**Fig. 3:**
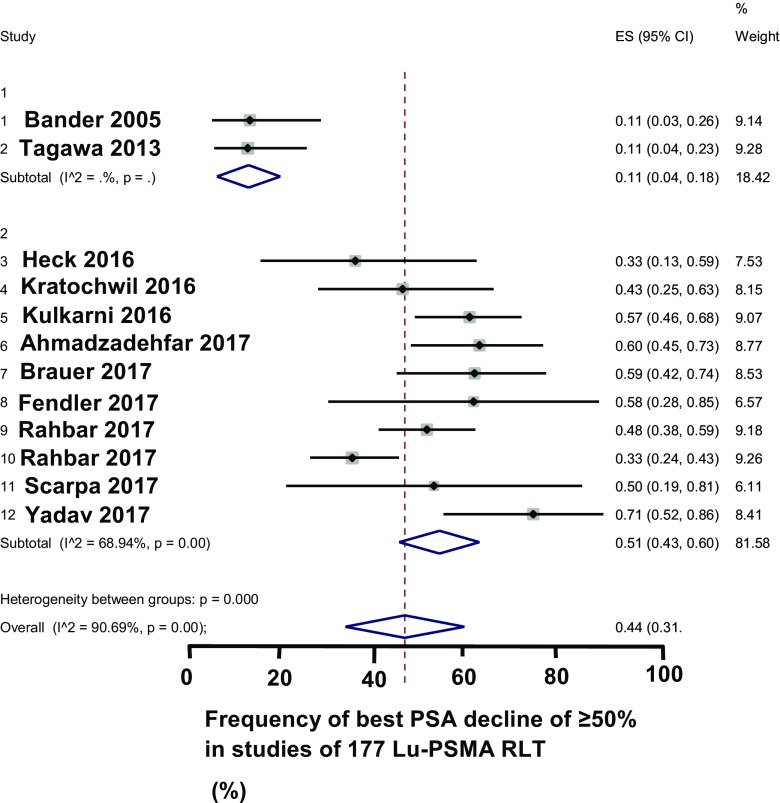
Forest plot showing the frequency of a best PSA decline of ≥50% in 12 articles with ^177^Lu-PSMA RLT, ^177^Lu-PSMA-617, and ^177^Lu-PSMA I&T (10 articles) gave a higher frequency of a best PSA decline of ≥50% than given by ^177^Lu-PSMA-J591 (2 articles). The forest plot shows pooled estimates of the frequency of a best PSA decline of ≥50% with 95% confidence intervals shown as a line

We undertook a funnel plot of the frequency of best PSA decline of ≥50% in the articles of ^177^Lu-PSMA RLT (Fig. 2a). The funnel plot did not indicate the articles had a publication bias. A forest plot of the articles summarized the frequency of best PSA decline of ≥50% for the two main types of ^177^Lu radioligands (Fig. 3). Overall, 44% [95% confidence intervals (CIs) 31–51%] of the patients had a best PSA decline of ≥50%. As for ^177^Lu-PSMA-617 RLT and ^177^Lu-PSMA I&T combined, 51% (95% CI: 43–60%) of the patients had a best PSA decline of ≥50%. Of evaluable articles, a median of 29% (IQR 8–36%) of the patients had objective remission. With an increasing number of cycles, the frequency of objective remission increased [37, 38]. In evaluable articles, the patients had a median overall survival of 14 months. Tagawa et al. [31] found median overall survival following treatment with ^177^Lu-J591 was 17 months. Ahmadzadefar et al. [35] found that patients with a best PSA decline of ≥50% after the third cycle of ^177^Lu-PSMA RLT lived significantly longer than patients with less extensive decline of PSA (17 months versus 10 months, *p* = 0.001, log-rank test). In evaluable articles, the mean of the median overall survival after ^177^Lu-PSMA RLT was 14 months. In two articles, overall survival remained above 50% during the follow-up [34, 37].

### Adverse effects

The three forms for ^177^Lu-PSMA RLT differed in adverse effects. Bander et al. [30] and Tagawa et al. [31] found that 10 of 35 patients and 21 of 47 patients, respectively, developed severe thrombocytopenia after treatment with ^177^Lu-J591. The patients were given platelet transfusions. Thus, 31 of 82 (39%) of the patients treated with ^177^Lu-J591 developed grade 3 or 4 thrombocytopenia in contrast to 6 of 243 (2%) of the patients treated with ^177^Lu-PSMA-617 and ^177^Lu-PSMA I&T (*p* < 0.001, χ^2^ test with one degree of freedom) [32, 33, 37, 38, 41]. Rahbar et al. [38] found that 8% of the patients treated with ^177^Lu-PSMA-617 RLT developed xerostomia.

### Studies of third-line treatment

A search for articles of third-line treatment gave 40 hits. The selected 16 articles included 1338 patients (Fig. 1b and Table 2) [24, 25, 42–55]. All articles reported retrospective cohort studies. The median of the median/mean age at diagnosis was 70 years (IQR 69–71 years). The median of the median/mean pretreatment PSA was 130 ng/ml (IQR 77–306 ng/ml). In all articles, docetaxel was the first systemic treatment. Patients with failure to respond docetaxel were treated with abiraterone, enzalutamide, and cabazitaxel. Most articles used abiraterone before enzalutamide, but a subgroup of patients in one article were given enzalutamide as the first AR pathway inhibitor [24]. Third-line treatment was abiraterone for 288 (21%) patients [24, 42, 43, 49, 50, 53], enzalutamide for 596 (45%) patients [24, 25, 48, 51, 52], and cabazitaxel for 454 (34%) patients [44–46, 48, 51, 54]. As for the third-line treatment, the dose for abiraterone was 1000 mg/day and 160 mg/day for enzalutamide. For cabazitaxel, the dose was 20 or 25 mg/m^2^ body surface intravenously every 3 weeks. Sella et al. [28] gave patients treated with cabazitaxel prophylaxis with granulocyte-colony stimulating factor (G-CSF).

**Table 2:** Findings in articles of third-line treatment (*n* = 16)

Author	No. of patients	Characteristics		Treatments		Outcome		
		Median age (yr)	Median pretreatment PSA (ng/ml)	Second-line treatment	Third-line treatment	Frequency of best PSA decline ≥50% (%)	Frequency of partial remission (%)	Median overall survival (months)
Azad [47]	68	72	NR	Abi	Enza	22 (15/68)	NR	11
Brasso [25]	137	71	348	Abi	Enza	18 (22/122)	12 (7/59)	8
Caffo [24]	49	75	NR	Abi	Enza	24 (12/49)	15 (7/49)	10
Cheng [48]	165	62	306	Abi	Enza	17 (28/165)	NR	12
Badrising [51]	102	NR	NR	Abi	Enza	25 (26/102)	NR	11
Davies [52]	34	69	52	Abi	Enza	NR	NR	10
De Bono [55]	69	70	71	Abi	Enza	28 (16/57)	NR	11
Loriot [42]	38	71	232	Enza	Abi	8 (3/38)	12 (1/12)	12
Noonan [43]	30	70	NR	Enza	Abi	3 (1/27)	NR	13
Caffo [24]	12	74	NR	Enza	Abi	25 (2/8)	15 (2/15)	15
Pezaro [44]	36	62	717	Abi	Caba	44 (16/36)	15 (3/20)	16
Sella [45]	24	65	128	Abi	Caba	33 (6/19)	15 (2/13)	8
Caffo [18]	94	71	NR	Abi	Caba	28 (27/94)	14 (13/94)	12
Wissing [50]	69	70	130	Abi	Caba	32 (21/66)	NR	15
Al Nakouzi [46]	79	69	307	Abi	Caba	35 (28/79)	NR	8
Kongsted [53]	66	68	NR	Abi	Caba	17 (11/66)	NR	12
Sonpavde [49]	36	69	77	Abi	Caba	NR	NR	12
Bando [54]	14	74	44	Abi	Caba	44 (6/14)	NR	12
Caffo [24]	16	71	NR	Enza	Caba	25 (4/16)	13 (2/16)	12
Bando [54]	20	74	44	Enza	Caba	45 (9/20)	NR	8
Caffo [24]	68		NR	Caba	Abi	24 (17/68)	15 (10/68)	12
Sonpavde [49]	77	68	112	Caba	Abi	NR	NR	18
Wissing [50]	63	66	291	Caba	Abi	18 (11/63)	NR	18
Kongsted [53]	25	NR	NR	Caba	Abi	68 (16/25)	NR	NR
Caffo [24]	21	70	NR	Caba	Enza	20 (4/21)	10 (5/21)	10
Total no.	1338							

All articles reported patients with mCRPC who had failed first-line treatment with docetaxel. Several articles reported more than one sequence of second- and third-line treatment.

Abbreviations: *Abi* abiraterone, *Caba* cabazitaxel, *Enza* enzalutamide, *NR* not reported

### Efficacy

**Fig. 4:**
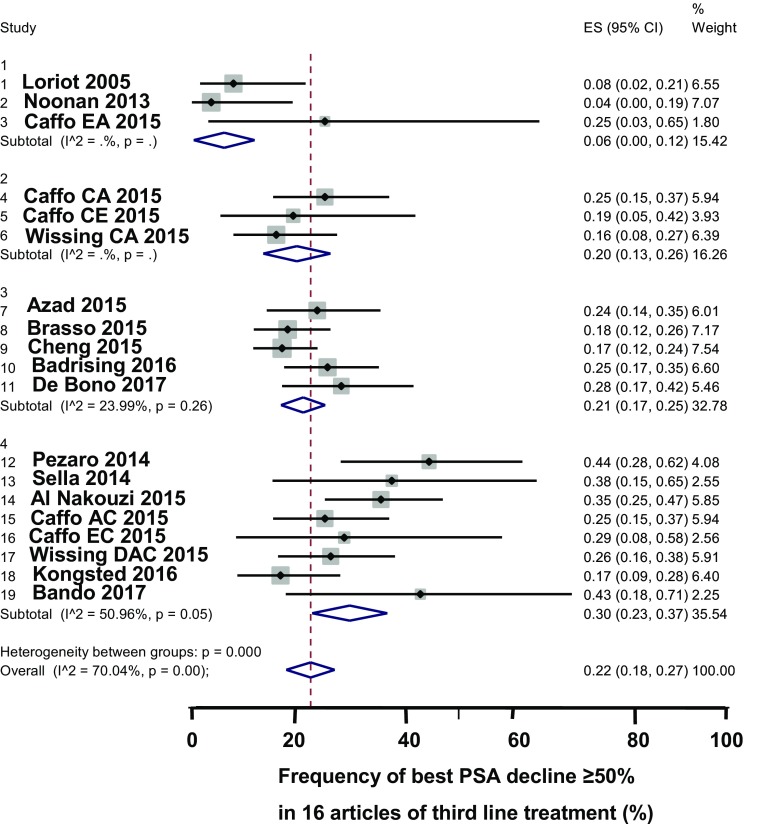
Forest plot regarding the frequency of a best PSA decline of ≥50% in 16 articles of third-line treatment. The frequency of a best PSA decline of ≥50% increased summarizing 3 articles with abiraterone, 5 articles with enzalutamide, and 8 articles with cabazitaxel. The forest plot shows pooled estimates of the frequency of best PSA decline of ≥50%, with the 95% confidence interval shown as a line

A funnel plot evaluated the articles of third-line treatment for the frequency of best PSA decline of ≥50% after the treatment. A funnel plot of cabazitaxel articles did not indicate publication bias (Fig. 2b). A forest plot shows the frequency of best PSA decline of ≥50% according to the sequences of second- and third-line drugs (Fig. 4). Summarizing the findings in all articles, 21% (95% CI 16–27%) of the patients had a PSA decline of ≥50%. After third-line treatment, the pooled frequency of best PSA decline of ≥50% was 4% with abiraterone, 20% with enzalutamide, and 29% with cabazitaxel. Hence, cabazitaxel caused a best decline of PSA of ≥50% more often than AR and AR signaling inhibitors (29% versus 19%, *p* = 0.001, χ^2^ test with one degree of freedom). In seven articles, the median frequency of objective remission was 15% (IQR 13–20%). Patients with a best PSA decline of ≥50% after treatment with enzalutamide lived longer than patients with a smaller best decline of PSA [25]. In evaluable articles, the median of the median overall survival was 11 months (range 7–20 months). An article showed that background clinical characteristics such a performance status, level of hemoglobin, and activity of serum alkaline phosphatase had a significant impact on overall survival.

### Adverse effects

The third-line drugs differed in adverse effects. Most articles did not report adverse effects. Loriot et al. [42] showed that 3 of 30 (10%) patients stopped treatment with enzalutamide due to adverse effects. Later, the patients were treated with abiraterone and it did not cause similar adverse effects. Kongsted et al. [53] found that 15 of 66 (23%) patients treated with cabazitaxel stopped the treatment due to adverse effects.

### Comparison of ^177^Lu-PSMA RLT and third-line treatment

**Fig. 5:**
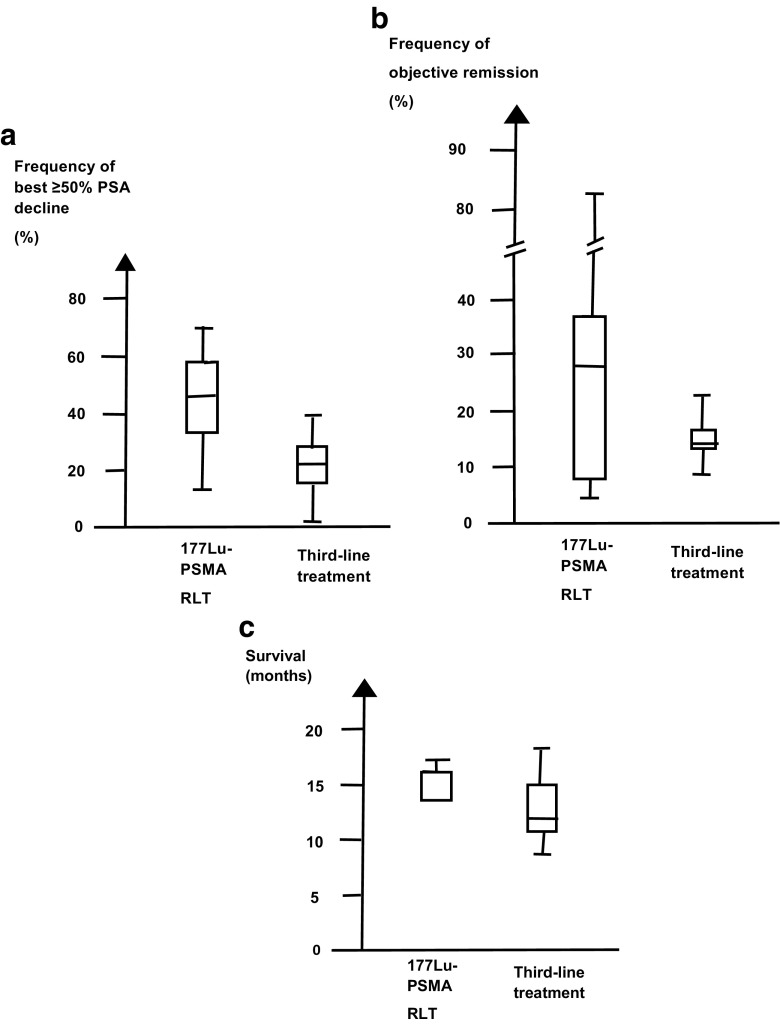
Boxplots show effect endpoints for 177Lu-PSMA RLT and third-line treatment. **a** Frequency of best PSA decline of ≥50%. **b** Frequency of objective remission. **c** Overall survival. The boxes show the 5, 50, and 75% percentiles. The whiskers show the full range

The articles of ^177^Lu-PSMA RLT and third-line treatment did not compare the outcomes head-to-head. Neither was ^177^Lu-PSMA RLT reported as third-line treatment. So, we could only indirectly compare ^177^Lu-PSMA RLT and third-line treatment. In the articles of the two types of treatment, the age at diagnosis was similar (the mean of the median age was 71 versus 69 years, *p* = 0.20, *t* test). Articles of ^177^Lu-PSMA RLT had higher pre-treatment PSA values than articles of third-line treatment, but the difference was not statistically significant (the mean of the median PSA level was 247 ml versus 197 ng/ml, *p* = 0.48, *t* test). Despite the similarities, ^177^Lu-PSMA RLT caused a best decline of PSA ≥50% twice as often as the third-line treatment (mean frequency 44% versus 22%, *p* = 0.0002, *t* test; Table 3). Figure 5a shows that the best PSA decline ≥50% differed between patients in the two groups of treatments. ^177^Lu-PSMA RLT also caused a higher frequency of objective remission than third-line treatment (Fig. 5b). Overall, 31 of 109 patients versus 43 of 275 patients had objective remission (*p* < 0.001, χ^2^ test with one degree of freedom). Figure 5c shows that patients given ^177^Lu-PSMA RLT tended to live longer than patients given third-line treatment (median of 14 months versus 11 months), but the difference was not statistically significant. Third-line treatment was stopped more often than ^177^Lu-PSMA RLT (22 of 66 patients versus 0 of 469 patients, *p* < 0.001, χ^2^ test with one degree of freedom).

**Table 3:** Endpoints for effect of treatment

Treatment		Endpoints		
		Frequency of best PSA decline ≥50% (%)	Frequency of objective remission (%)	Overall survival (months)
^177^Lu PSMA RLT		49	28.5	14
Third-line treatment		22	15	12
Third-line treatment with abiraterone	Second-line enzalutamide	7	8.3	13
	Second-line cabazitaxel	22	14	18
Third-line treatment with enzalutamide1		19	17	11
Third-line treatment with cabazitaxel		31.5	15	12

The table shows the median value for the endpoints in the articles according to the treatment or the treatment sequence.

## Discussion

In our comparisons, up to half the patients given ^177^Lu-PSMA RLT obtained a best PSA decline of ≥50%, whereas up to a third of the patients given third-line treatment obtained such a decline of PSA. Specifically, ^177^Lu-PSMA-617 RLT and ^177^Lu-PSMA I&T reduced PSA ≥50% more often and caused fewer adverse effects than cabazitaxel.

Our literature search for ^177^Lu-PSMA RLT and third-line treatment differed because different specialists gave the treatments to different groups of patients. But we undertook similar statistical analyses of clinical characteristics and outcomes for both types of treatments in our meta-analysis of the selected articles. Funnel plots did not indicate publication bias in the articles regarding the two types of treatment. The forest plots gave robust findings regarding ^177^Lu-J591, and third-line therapy with abiraterone and enzalutamide.

Our systematic review found a higher response rate for ^177^Lu-PSMA RLT than the previous systematic review by Calopedos et al. [16]. The difference may be due to differences in selection of articles. Our systematic review included more recent articles than the previous review and excluded data reported only as abstracts. Furthermore, our systematic review evaluated the selection of articles with funnel plots, and included boxplots of the frequency of objective remission and overall survival as end-points for effect of the treatment. Anyway, both systematic review supported that ^177^Lu-PSMA RLT is effective as treatment of mCRPC [16]. ^177^Lu-PSMA-617 RLT and ^177^Lu-PSMA I&T caused a best PSA decline of ≥50% more often and caused less adverse effects than ^177^Lu-J591. Articles of ^177^Lu-PSMA-617 RLT and ^177^Lu-PSMA I&T reported mainly transitory adverse effects. Reviews of dosimetry and practical aspects indicated that ^177^Lu-PSMA RLT was safe [13, 56]. Typically, for the articles in our review, ^177^Lu-PSMA RLT was administered at 8-week intervals with an activity of 6 GBq of ^177^Lu for each cycle. The typical intervals and ^177^Lu activities were in accordance with recommendations by the German Society of Nuclear Medicine [57]. However, the low number of cycles for two thirds of the patients was not optimal, and neither was the recommended interval and activity for ^177^Lu-PSMA-RLT. A recent trial escalated the activity for each cycle from 6 to 9.3 GBq [58]. An Austrian study gave three cycles of 7.4 GBq with 4-week intervals. Also, a third study proposed a shorter interval between cycles [40]. An Australian trial gave an ^177^Lu activity of 4–8 GBq for each cycle and used 6-week intervals between the cycles (ACTRN12615000912583), presented as an abstract for the ESMO conference 2017 (Abstract 7850, Ann Oncol 2017, 28 suppl 5, v269–v294).

As for third-line treatment, our systematic review reported poorer overall survival than the review by Maines et al. [22]. The previous review summarized survival after failure to first-line docetaxel, whereas our review summarized survival from start of third-line treatment. The systematic review and meta-analysis by Maines et al. also differed with an Italian multicenter study regarding the impact cabazitaxel given as second- or third-line treatment had on overall survival [22, 24]. Most specialist at APCCC 2017 voted for cabazitaxel as third-line treatment. In contrast in our review, nearly half the reported patients had enzalutamide as third-line treatment and cabazitaxel was third-line treatment only for a third of the patients. As for patients given abiraterone as third-line treatment, the outcome suggested a cross-resistance after second-line treatment with enzalutamide.

Given as third-line treatment, cabazitaxel reduced the best PSA decline of ≥50% more often than abiraterone and enzalutamide, but caused more adverse effects and did not increase overall survival.

The third-line treatment in our articles followed general practice. All articles used a sequence of monotherapies with docetaxel as the first systemic treatment. All articles used standard dose for the third-line drugs. Today, cabazitaxel may cause less adverse effects than those reported following cabazitaxel in our review. Trials comparing 20-mg/m^2^ and 25-mg/m^2^ body surface dose levels of cabazitaxel showed non-inferiority for the low dose [59]. Accordingly, APCCC 2017 preferred the low dose of cabazitaxel combined with G-CSF prophylaxis from the start of treatment [12]. Thus, the articles of third-line treatment pointed to the real effect of the treatment.

The systematic review of Calopedos et al. [16] compared the outcome following ^177^Lu-PSMA RLT with the outcome following cabazitaxel as reported in the TROPIC trial [4]. In contrast, we compared articles of cohort studies of ^177^Lu-PSMA RLT and of third-line treatment using the same statistical methodology. Our comparison might give a more realistic estimate of the difference between the two types of treatment. Our findings contradicted conventional assumptions of inferiority or non-inferiority for ^177^Lu-PSMA RLT compared with third-line treatment. APCCC 2017 recommended third-line treatment only with drugs known to prolong life as second-line treatment [12]. But in our review, ^177^Lu-PSMA RLT was more effective than third-line treatment despite being given later in the treatment sequence for mCRPC. Correspondingly, ^177^Lu-PSMA-617 RLT gave better PSA decline than abiraterone in a recent case report [18].

Our systematic review is a correlate to APCCC 2017 [12, 19]. For glu-ureido-based inhibitor ^177^Lu-PSMA RLT, our evidence was articles including 582 patients from 9 centers. Our evidence for cabazitaxel as third-line treatment was articles including 454 patients from 9 centers. We find it irrational that APCCC 2017 insisted that the effect of ^177^Lu-PSMA RLT should be proven in a randomized trial whereas the APCCC 2017 recommended third-line treatment without such a proof of effect. Evidence-based medicine prefers to base treatment decisions on a systematic review as alternative to the opinion of the (medical oncology) experts. In absence of randomized trials, oncologists should choose between ^177^Lu-PSMA-617 RLT and third-line treatment based on effects and adverse effects of the treatments [20]. APCCC 2017 voted that patients with end-stage mCRPC should be treated with carboplatin-containing regimens [12]. However, information regarding carboplatin-containing regimens is sparse. A preference for carboplatin-containing regimens for end-stage mCRPC implies that the PC has small cell/neuroendocrine histology. But the assumption has not been proven.

ClinicalTrials.gov registered ongoing studies of ^177^Lu-PSMA RLT (NCT03042468, NCT03042312,). Twenty-five ongoing studies are evaluating the aspects of third-line treatment [37]. ClinicalTrials.gov also registered five studies of third-line treatment (NCT02729103, NCT01718353, NCT02254785, NCT02485691, and NCT02125357). Ongoing studies aim to define the best schedule for ^177^Lu-PSMA RLT. The study NCT03042468 is evaluating dose escalation of ^177^Lu activity from 1.85 to 11.6 GBq for each cycle, given at 2-week intervals. A German study reported dose escalation of ^177^Lu activity for each cycle from 4 to 9.3 GBq [58]. Other studies examine new roles for ^177^Lu-PSMA RLT. An ongoing study is evaluating ^177^Lu-PSMA RLT for patients with lymph node metastatic CRPC. Similarly, APCCC 2017 argued for trials that compare ^177^Lu-PSMA RLT and third-line treatment [19]. ^177^Lu-PSMA RLT is also being examined as part of combination therapy. A case report described outcome following treatment with a combination of ^177^Lu-PSMA-617 RLT and EBRT [60]. An ongoing study (NCT00916123) is evaluating ^177^Lu-J591 combined with docetaxel.

In recent years, management of PC has changed rapidly [11, 61, 62]. AR and AR signaling inhibitors cause less adverse effects than docetaxel. Therefore, today, AR and AR signaling inhibitors may be the first systemic treatment of castration-naïve mPC and mCRPC [63–67]. Initiation of androgen deprivation therapy (ADT) may also be combined with docetaxel [53, 54]. Thus, our analyses of post-docetaxel treatment remain relevant for patients with CRPC today where initiation of ADT is combined with docetaxel.

Our systematic review and meta-analysis has limitations. The articles mainly used ^177^Lu-PSMA RLT for patients with end-stage PC. So, the review did not evaluate the efficacy for patients in an earlier phase of PC. Background factors may be important for overall survival after third-line treatment, but the articles rarely reported these characteristics. The articles summarized overall survival from start of ^177^Lu-PSMA RLT or start of third-line treatment and not from a common point in the progression of the disease such as the diagnosis of mCRPC. As the articles had a short follow-up, our review did not assess long-term effects and adverse effects of ^177^Lu-PSMA RLT.

## Conclusion

^177^Lu-PSMA RLT had better effects and caused less adverse effects than third-line treatment.
